# Efficacy and safety of once-daily NVA237 in patients with moderate-to-severe COPD: the GLOW1 trial

**DOI:** 10.1186/1465-9921-12-156

**Published:** 2011-12-07

**Authors:** Anthony D'Urzo, Gary T Ferguson, Jan A van Noord, Kazuto Hirata, Carmen Martin, Rachael Horton, Yimeng Lu, Donald Banerji, Tim Overend

**Affiliations:** 1 Department of Family and Community Medicine (DFCM), University of Toronto, Ontario, Canada; 2 Pulmonary Research Institute of Southeast Michigan, Livonia, Michigan, USA; 3 Atrium Medisch Centrum, Heerlen, The Netherlands; 4 Osaka City University, Abeno-ku, Osaka, Japan; 5 Novartis Horsham Research Centre, West Sussex, UK; 6 Novartis Pharmaceuticals Corporation, East Hanover, NJ, USA

**Keywords:** NVA237, once-daily, COPD, LAMA, dyspnoea, quality of life, exacerbations

## Abstract

**Background:**

NVA237 is a once-daily dry-powder formulation of the long-acting muscarinic antagonist glycopyrronium bromide in development for the treatment of chronic obstructive pulmonary disease (COPD). The glycopyrronium bromide in COPD airways clinical study 1 (GLOW1) evaluated the efficacy, safety and tolerability of NVA237 in patients with moderate-to-severe COPD.

**Methods:**

Patients with COPD with a smoking history of ≥ 10 pack-years, post-bronchodilator forced expiratory volume in 1 second (FEV_1_) < 80% and ≥ 30% predicted normal and FEV_1_/forced vital capacity < 0.70 were enrolled. Patients were randomized to double-blind treatment with NVA237 50 μg once daily or placebo for 26 weeks with inhaled/intranasal corticosteroids or H_1 _antagonists permitted in patients stabilized on them prior to study entry. The primary outcome measure was trough FEV_1 _at Week 12.

**Results:**

A total of 822 patients were randomized to NVA237 (n = 552) or placebo (n = 270). Least squares mean (± standard error) trough FEV_1 _at Week 12 was significantly higher in patients receiving NVA237 (1.408 ± 0.0105 L), versus placebo (1.301 ± 0.0137 L; treatment difference 108 ± 14.8 mL, p < 0.001). Significant improvements in trough FEV_1 _were apparent at the end of Day 1 and sustained through Week 26. FEV_1 _was significantly improved in the NVA237 group versus placebo throughout the 24-hour periods on Day 1 and at Weeks 12 and 26, and at all other visits and timepoints. Transition dyspnoea index focal scores and St. George's Respiratory Questionnaire scores were significantly improved with NVA237 versus placebo at Week 26, with treatment differences of 1.04 (p < 0.001) and-2.81 (p = 0.004), respectively. NVA237 significantly reduced the risk of first moderate/severe COPD exacerbation by 31% (p = 0.023) and use of rescue medication by 0.46 puffs per day (p = 0.005), versus placebo. NVA237 was well tolerated and had an acceptable safety profile, with a low frequency of cardiac and typical antimuscarinic adverse effects.

**Conclusions:**

Once-daily NVA237 was safe and well tolerated and provided rapid, sustained improvements in lung function, improvements in dyspnoea, and health-related quality of life, and reduced the risk of exacerbations and the use of rescue medication.

**Trial registration:**

ClinicalTrials.gov: NCT01005901

## Introduction

Chronic obstructive pulmonary disease (COPD) is characterized by progressive airflow limitation, results in breathlessness and reduced exercise capacity, and is a leading cause of morbidity and mortality [1,2]. The main goals of pharmacotherapy are to prevent and control symptoms, reduce the frequency and severity of exacerbations, improve health status, and increase exercise tolerance [1]. Symptomatic treatment relies to a large extent on the use of bronchodilators [1], including long-acting muscarinic antagonists (LAMAs).

Tiotropium is the most frequently used LAMA worldwide and is an effective bronchodilator. Some patients taking tiotropium may experience adverse events (AEs) such as dry mouth, urinary problems and constipation [3]. Tiotropium has a slow onset of action, with peak effects on lung function achieved after up to 3 hours [4]. NVA237 is a once-daily dry-powder formulation of the LAMA glycopyrronium bromide that is currently in development for the treatment of COPD. In common with other LAMAs, the bronchodilatory effects of NVA237 result from blockade of muscarinic type 1 (M1) and type 3 (M3) receptors, which are involved in transmission of nerve impulses (M1) and promotion of contraction (M3) in airway smooth muscle [5].

Among patients with moderate-to-severe COPD, once-daily NVA237 provides sustained 24-hour bronchodilation, has a rapid onset of action and is safe and well tolerated [6-8]. All evaluated doses of NVA237 (up to 200 μg) were well tolerated, with doses of 50 or 100 μg once daily having greater efficacy than lower doses [6-8].

The objective of the Phase III glycopyrronium bromide in COPD airways clinical study 1 (GLOW1) was to evaluate the efficacy, safety and tolerability of once-daily NVA237 50 μg, compared with placebo, in patients with moderate-to-severe COPD.

## Methods

### Patients

Men and women with moderate-to-severe COPD (as defined in the 2008 GOLD guidelines) [9] were eligible for enrolment if they were ≥ 40 years of age, had a smoking history of ≥ 10 pack-years, post-bronchodilator forced expiratory volume in 1 second (FEV_1_) of < 80% and ≥ 30% of predicted normal value and post-bronchodilator FEV_1_/forced vital capacity (FVC) ratio of < 0.70. Exclusion criteria included lower respiratory tract infection within 6 weeks, concomitant pulmonary disease, history of asthma, lung cancer or long QT syndrome or QTc > 450 ms (males) or > 470 (females), symptomatic prostatic hyperplasia, bladder-neck obstruction, moderate/severe renal impairment, urinary retention, narrow-angle glaucoma and history of alpha-1 antitrypsin deficiency. Patients were also excluded if they were participating in a supervised pulmonary rehabilitation programme, had contraindications for tiotropium or ipratropium or had experienced adverse reactions to inhaled anticholinergics.

All patients gave written, informed consent to participate in the study, which was conducted according to the principles of Good Clinical Practice and the Declaration of Helsinki [10]. The study protocol (study NCT01005901) was reviewed and approved by institutional review boards and ethics committees at participating centres.

### Study design and treatments

In this double-blind, placebo-controlled study, patients who completed a 7-day pre-screening period and a subsequent 14-day run-in period, were randomized in a 2:1 ratio to 26 weeks of treatment with NVA237 50 μg once daily or placebo administered via a low-resistance single-dose dry-powder inhaler (SDDPI; Breezhaler^®^). In addition to the study treatment, concomitant medications (inhaled corticosteroids [ICSs], intranasal corticosteroids or H_1 _antagonists) were permitted in patients who had been stabilized on a recommended and constant dose prior to study entry. Patients were required to cease taking long-acting bronchodilator therapy before beginning the run-in period (with a 48-hour washout period for long-acting β_2_-agonist [LABA]/ICS combinations and a 7-day washout period for tiotropium) and were instructed to use rescue medication. Patients receiving LABA/ICS combinations were switched to an equivalent dose of the ICS contained in the fixed-dose combination product, with rescue medication available if required. ICS doses had to remain stable during screening (patients failing screening for this reason could be re-screened if the ICS dose was stabilized for 1 month). Patients previously treated with a single-agent ICS continued on their pre-study regimen. During the randomized treatment period all patients continued to receive the same ICS regimen that they received during screening. Patients were provided with a salbutamol/albuterol inhaler to use as rescue medication throughout the study.

### Efficacy assessments

Efficacy was based on centralized spirometry and assessed in the full analysis set (FAS), which included all randomized patients who received at least one dose of study drug; patients were analyzed according to the treatment to which they were randomized. Pulmonary function was assessed in accordance with American Thoracic Society/European Respiratory Society standards [11], and the spirometry data was reviewed by a pulmonologist to ensure data quality. To reduce variability, the same equipment was used for all measurements and, whenever possible, the same staff member evaluated and coached each patient throughout the study. The spirometer was calibrated every morning before taking measurements. The primary outcome measure was trough FEV_1 _(mean of the values at 23 h 15 min and 23 h 45 min after dosing) at Week 12. Key secondary outcome measures were breathlessness on the transition dyspnoea index (TDI) and health-related quality of life (HRQoL) according to the St. George's Respiratory Questionnaire (SGRQ) at Week 26, while important secondary outcomes were time to first moderate or severe COPD exacerbation and mean daily rescue medication use over 26 weeks.

At Visit 2, all patients were provided with an electronic patient diary to record morning and evening daily clinical symptoms: cough, wheezing, shortness of breath, sputum volume and colour, night time awakenings and rescue medication use. Designated investigator site staff determined exacerbations by reviewing diary card data with the patient. COPD exacerbations were defined as worsening of two or more major symptoms (dyspnoea, sputum volume or sputum purulence) for at least 2 consecutive days or worsening of any one major symptom together with any minor symptom (colds, fever without other cause, increased cough, increased wheeze or sore throat) for at least 2 consecutive days. Exacerbations were considered to be of moderate severity if they required treatment with systemic corticosteroids or an antibiotic and were considered severe if they also required hospitalization [12,13]. Patients experiencing an exacerbation were expected to continue in the study if, in the opinion of the investigator, they could safely be returned to their pre-exacerbation concomitant medications. Patients requiring addition of new concomitant COPD medications after an exacerbation or who were receiving intra-muscular depot corticosteroids were withdrawn from the study.

Other efficacy endpoints included trough FEV_1 _at the end of Day 1 and at Week 26, serial spirometry on Day 1 and at Weeks 12 and 26, and inspiratory capacity (IC) on Day 1 and at Weeks 12 and 26. Serial spirometry was performed in a subset of the FAS, with measurements made over a 12-hour period on Day 1 and during 24 hours at Weeks 12 and 26. Patients practiced the measurement of IC at screening until reproducible results could be obtained. Before undertaking an IC measurement, patients performed normal tidal breathing and then inhaled to their maximum while receiving verbal encouragement.

### Safety assessments

The safety analysis population included all patients who received at least one dose of study drug, regardless of whether they were randomized. Patients were analyzed according to the treatment they received, irrespective of whether this was the treatment to which they were randomized. Safety was assessed by recording of treatment-emergent AEs and monitoring of vital signs, electrocardiograms and laboratory analyses. AEs were coded using the Medical Dictionary of Regulatory Activities (MedDRA) and summarized by primary system organ class, preferred term, severity and relationship to study drug.

### Statistics

The primary outcome (trough FEV_1 _at Week 12) was analyzed using a mixed model, with treatment as a fixed effect and baseline FEV_1_, ICS use and FEV_1 _reversibility in response to ipratropium as covariates. To reflect the randomization scheme, the model also included baseline smoking status (current/ex-smoker) and region as fixed effects with centre nested within region as a random effect. TDI and SGRQ scores and rescue medication use were analyzed using the same mixed model specified for the primary analysis, with baseline FEV_1 _replaced as a covariate by baseline dyspnoea index, baseline SGRQ score, and baseline daily rescue medication use, respectively.

Time to the first moderate or severe COPD exacerbation was analyzed using a Cox regression model, including terms for treatment, baseline ICS use, baseline daily total symptom score, number of moderate or severe COPD exacerbations in the year prior to screening, FEV_1 _reversibility, baseline smoking status and region. Patients who withdrew from the study and did not experience a moderate or severe COPD exacerbation were censored at the date of the last visit or last dose of study medication (whichever was later). Patients who completed the study and did not experience a moderate or severe COPD exacerbation were censored at the completion visit date. The event rate of moderate or severe COPD exacerbation was analyzed by a negative binomial model.

A fixed sequence test procedure was used to handle multiplicity, with superiorities of NVA237 over placebo tested sequentially in three families (primary outcome; key secondary outcomes; important secondary outcomes) using a hierarchical procedure with Hochberg step up adjustment and type one error rate controlled at the 0.05 level within each family. To proceed to the next family of tests in the hierarchy, tests in the previous families had to be statistically significant at the type I error rate of 0.05 after applying the Hochberg step up adjustment. This fixed sequence testing procedure had no impact on the testing of other secondary variables.

For the primary efficacy analysis (trough FEV_1 _at Week 12), values taken within 6 hours of rescue medication use or 7 days of systemic corticosteroid use were excluded. The last observation of pre-dose trough FEV_1 _was carried forward (LOCF) for missing values. Similarly, missing values for the assessments of trough FEV_1 _at Week 26 were also imputed using the LOCF (values of pre-dose trough FEV_1 _were not carried forward for more than 11 weeks).

## Results

### Patient disposition and baseline characteristics

**Figure 1:**
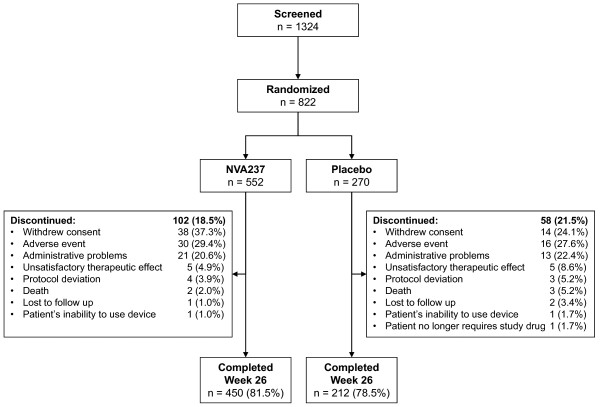
**Patient disposition**.

A total of 822 patients were randomized to treatment with NVA237 (n = 552) or placebo (n = 270). Approximately 80% of patients completed the study (Figure 1). A higher percentage of discontinuations were due to withdrawal of consent in the NVA237 group (37.3%) than in the placebo group (24.1%) and a higher percentage were due to unsatisfactory therapeutic effect in the placebo group (8.6%) than in the NVA237 group (4.9%) (Figure 1). Baseline demographics and clinical characteristics were well balanced between treatment groups (Table 1).

**Table 1:** Demographic and baseline clinical characteristics

	NVA237 50 μg (n = 550)	Placebo (n = 267)
Mean (SD) age, years [range]	63.8 (9.47) [40.0-91.0]	64.0 (8.96) [42.0-85.0]
Male gender, n (%)	454 (82.5)	215 (80.5)
Ethnicity, n (%)		
Caucasian	346 (62.9)	166 (62.2)
Asian	195 (35.5)	94 (35.2)
Black	3 (0.5)	3 (1.1)
Other	6 (1.1)	4 (1.4)
Mean (SD) body mass index, kg/m^2^	25.8 (5.88)	25.6 (5.60)
Severity of COPD, n (%)		
Moderate	331 (60.2)	166 (62.2)
Severe	217 (39.5)	99 (37.1)
Very severe	2 (0.4)	2 (0.7)
Mean (SD) duration of COPD, years	5.87 (5.798)	6.49 (6.790)
Baseline COPD exacerbation history*, n (%)		
0 exacerbations	433 (78.7)	210 (78.7)
1 exacerbation	90 (16.4)	43 (16.1)
≥ 2 exacerbations	27 (4.9)	14 (5.2)
ICS use at baseline, n (%)	301 (54.7)	136 (50.9)
Smoking history, n (%)		
Ex-smoker	370 (67.3)	176 (65.9)
Current Smoker	180 (32.7)	91 (34.1)
Mean (SD) duration of smoking, pack years Mean (SD) FEV_1 _(L) pre-bronchodilator Mean (SD) FEV_1 _(L) post-bronchodilator	44.9 (28.08) 1.34 (0.45) 1.49 (0.46)	44.6 (24.80) 1.28 (0.43) 1.45 (0.45)
Mean (SD) post-bronchodilator FEV_1 _percentage predicted	54.75 (13.05)	54.33 (12.84)
Mean (SD) post-bronchodilator FEV_1 _reversibility, (%)	13.0 (14.21)	15.05 (13.70)
Mean (SD) post-bronchodilator FEV_1_/FVC, (%)	50.15 (10.26)	49.92 (10.22)

*The number of moderate or severe COPD exacerbations in the year prior to screening

SD: standard deviation

### Efficacy

**Figure 2:**
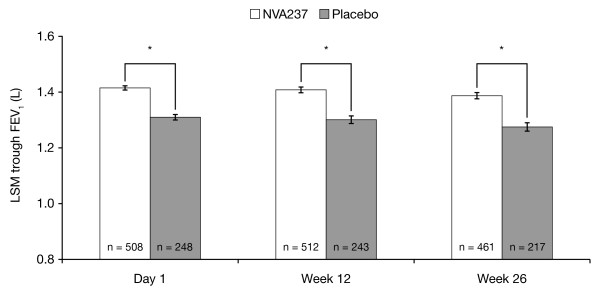
**Trough FEV_1 _on Day 1 and at Weeks 12 and 26**. Data are least squares means ± standard error; *p < 0.001; Treatment differences: Day 1 = 105 ± 10.9 mL; Week 12 = 108 ± 14.8 mL; Week 26 = 113 ± 16.5 mL.

Least squares mean (LSM) [± standard error (SE)] trough FEV_1 _at Week 12 was significantly higher in patients receiving NVA237 (1.408 ± 0.0105 L) compared with placebo (1.301 ± 0.0137 L), with LSM treatment difference of 108 ± 14.8 mL (p < 0.001; Figure 2). Trough FEV_1 _was also significantly higher in the NVA237 group at the end of Day 1 (1.414 ± 0.0075 L) and at Week 26 (1.387 ± 0.0112 L), compared with placebo (1.309 ± 0.0099 L and 1.275 ± 0.0150 L, respectively; both p < 0.001; Figure 2). The treatment difference was 105 ± 10.9 mL at the end of Day 1 and 113 ± 16.5 mL at Week 26 (both p < 0.001).

**Figure 3:**
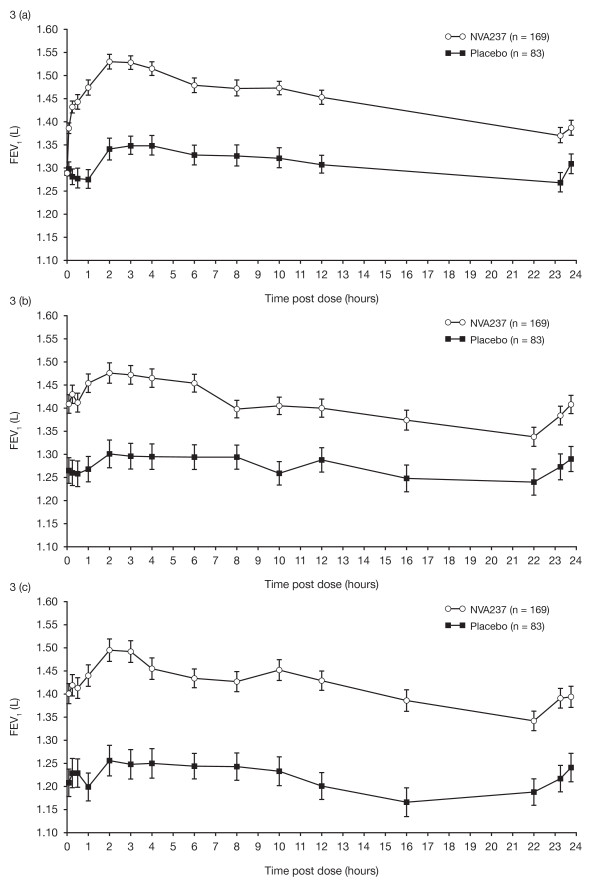
**Serial spirometry results on Day 1 (a) and at Weeks 12 (b) and 26 (c)**. All timepoints are statistically significant (p < 0.01).

On Day 1, LSM (± SE) difference in FEV_1 _between NVA237 and placebo was 93 ± 8.5 mL at 5 minutes and 144 ± 9.7 mL at 15 minutes (both p < 0.001). IC was also significantly higher in patients in the FAS population receiving NVA237, at all timepoints on Day 1 and at Weeks 12 and 26 (all p < 0.001; Table 2). The NVA237-placebo differences for IC by the end of Day 1, Week 12 and Week 26 were 104 mL, 97 mL and 113 mL, respectively (all p < 0.001). Serial spirometry in a subpopulation of patients revealed significantly higher values of FEV_1 _at all timepoints throughout the 24-hour periods on Day 1 and at Weeks 12 and 26 in patients receiving NVA237 compared with placebo (Figure 3).

**Table 2:** Inspiratory capacity on Day 1 and at Weeks 12 and 26 in the FAS population

	Inspiratory capacity, L			p-value
		
	NVA237 50 μg (n = 534)	Placebo (n = 260)	Difference	
Day 1				
25 min	2.110 ± 0.0148	1.930 ± 0.0194	0.181 ± 0.0212	< 0.001
1 h 55 min	2.166 ± 0.0157	1.978 ± 0.0210	0.189 ± 0.0230	< 0.001
3 h 55 min	2.139 ± 0.0164	1.970 ± 0.0217	0.169 ± 0.0232	< 0.001
23 h 40 min	2.019 ± 0.0168	1.915 ± 0.0222	0.104 ± 0.0239	< 0.001
Week 12				
-20 min	1.967 ± 0.0221	1.883 ± 0.0281	0.084 ± 0.0286	0.003
25 min	2.048 ± 0.0216	1.900 ± 0.0274	0.148 ± 0.0278	< 0.001
1 h 55 min	2.103 ± 0.0232	1.923 ± 0.0297	0.180 ± 0.0305	< 0.001
3 h 55 min	2.040 ± 0.0218	1.898 ± 0.0285	0.142 ± 0.0300	< 0.001
23 h 40 min	2.009 ± 0.0212	1.912 ± 0.0282	0.097 ± 0.0296	< 0.001
Week 26				
-20 min	1.988 ± 0.0202	1.879 ± 0.0267	0.109 ± 0.0281	< 0.001
25 min	2.055 ± 0.0205	1.905 ± 0.0272	0.150 ± 0.0285	< 0.001
1 h 55 min	2.107 ± 0.0214	1.929 ± 0.0288	0.178 ± 0.0309	< 0.001
3 h 55 min	2.080 ± 0.0206	1.915 ± 0.0283	0.165 ± 0.0304	< 0.001
23 h 40 min	1.997 ± 0.0192	1.884 ± 0.0257	0.113 ± 0.0275	< 0.001

Values are least squares means ± standard errors

**Figure 4:**
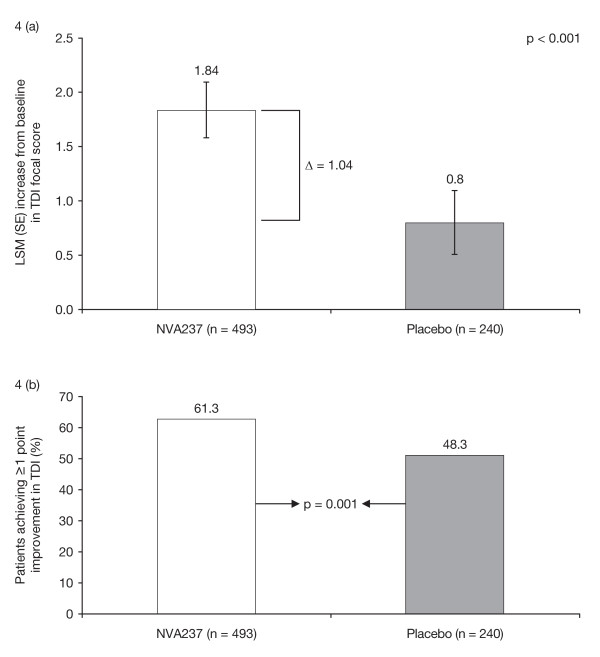
**Dyspnoea on the Transition Dyspnoea Index at Week 26 (a) TDI focal score at Week 26 (b) Patients achieving ≥ 1 point improvement in TDI at Week 26**. TDI = Transition Dyspnoea Index (≥ 1 point is the minimum clinically important difference [MCID]).

Patients receiving NVA237 had a significantly (p < 0.001) greater TDI focal score at Week 26 (1.84) compared with placebo (0.80), with a treatment difference of 1.04, which exceeded the 1 point treatment difference considered as clinically important [14] (Table 3, Figure 4a). In addition, patients receiving NVA237 were 1.7-fold more likely to have a clinically meaningful (≥ 1 point) improvement in TDI focal score compared with placebo (p = 0.001; Figure 4b). Similarly, at Week 12, the proportion of patients with a clinically meaningful improvement in TDI focal score was greater in the NVA237 group than in the placebo group (p = 0.013). Patients receiving NVA237 also had a significantly (p = 0.004) lower (better) SGRQ score at Week 26 (39.50) than those receiving placebo (42.31), with a treatment difference of -2.81, which was statistically significant but did not reach the threshold for clinical relevance (≥ 4 point reduction) [15] (Table 3). The percentage of patients achieving a clinically meaningful improvement in SGRQ was significantly higher with NVA237 than with placebo (56.8% versus 46.3%; odds ratio [OR] 1.58, 95% confidence interval [CI] 1.138-2.196; p = 0.006).

**Table 3:** Dyspnoea, health-related quality of life and use of rescue medication up to Week 26

	NVA237 50 μg	Placebo
TDI focal score		
Least squares mean (± SE)	1.84 ± 0.257	0.80 ± 0.294
Least squares mean difference (± SE)	1.04 ± 0.235	
p-value	< 0.001	
SGRQ score		
Baseline mean	46.11	46.34
Least squares mean (± SE)	39.50 ± 0.813	42.31 ± 0.992
Least squares mean difference (± SE)	-2.81 ± 0.961	
p-value	0.004	
Rescue medication use, puffs/day		
LSM change from baseline (± SE)	-1.21 ± 0.122	-0.75 ± 0.156
Least squares mean difference (± SE)	-0.46 ± 0.164	
p-value	0.005	

TDI: transition dyspnoea index, SGRQ: St. George's Respiratory Questionnaire

**Figure 5:**
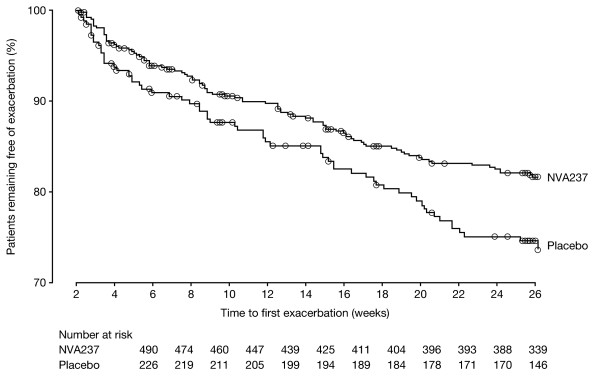
**Kaplan-Meier plot of the time to first moderate or severe COPD exacerbation**.

NVA237 significantly reduced the risk of COPD exacerbations in terms of time to first moderate or severe COPD exacerbation by 31% compared with placebo (hazard ratio [HR] 0.69, 95% CI 0.500-0.949; p = 0.023; Figure 5). During the 26-week study, 93 of 532 patients (17.5%) in the NVA237 group had one or more moderate or severe COPD exacerbation, compared with 63 of 260 patients (24.2%) in the placebo group. Further, there was a significant reduction in the risk of severe COPD exacerbations leading to hospitalization in the NVA237 group versus placebo (HR 0.35, 95% CI 0.141-0.857; p = 0.022). A significant reduction in the percentage of hospitalizations due to COPD exacerbations (1.7% versus 4.2%, OR 0.34, 95% CI 0.129-0.868; p = 0.024) and a numerical reduction in the rate of moderate or severe exacerbations with NVA237 versus placebo (0.43 versus 0.59/year; rate ratio 0.72; p = 0.071) was also observed in the NVA237 group versus placebo. The use of rescue medication was significantly (p = 0.005) lower in patients receiving NVA237 than in those receiving placebo, with a between-group difference of 0.46 puffs/day (Table 3).

### Safety

The incidence of AEs was lower in patients receiving NVA237 (57.5% of patients), compared with placebo (65.2%), largely due to a higher frequency of COPD worsening in the placebo group (Table 3). Other AEs, including those typically associated with anticholinergics (gastrointestinal disturbances, urinary difficulty, urinary retention, dry mouth), occurred at low frequencies in the NVA237 and placebo groups.

Serious adverse events (SAEs) and discontinuations due to AEs were less frequent in patients receiving NVA237 than in those receiving placebo (Table 4). With the exception of serious COPD worsening (less frequent in the NVA237 group), the majority of individual SAEs occurred in similar percentages of patients in the NVA237 and placebo groups. In the NVA237 group there were three serious atrial fibrillation (AF) events, two of which occurred in patients with a medical history of AF (neither was considered related to study treatment) and one of which occurred in a patient with a history of myocardial ischaemia but no history of AF (the event was suspected to be related to study treatment). Two patients receiving NVA237 had serious congestive heart failure events, one of whom had no relevant medical history related to the condition and one of whom had a prior diagnosis of AF. None of the cases of congestive heart failure events was considered to be related to study medication.

**Table 4:** Frequency of adverse events occurring in ≥ 3% of patients in either group, serious adverse events occurring in two or more patients in either group, deaths, discontinuations due to adverse events and electrocardiographic abnormalities

	NVA237 50 μg (n = 550)	Placebo (n = 267)
Patients with adverse events, n (%)	317 (57.5)	174 (65.2)
COPD worsening	111 (20.2)	73 (27.3)
Nasopharyngitis	28 (5.1)	21 (7.9)
Cough	26 (4.7)	13 (4.9)
Upper respiratory tract infection	23 (4.2)	20 (7.5)
Dyspnoea	18 (3.3)	10 (3.7)
Pyrexia	17 (3.1)	13 (4.9)
Upper respiratory tract infection, bacterial	17 (3.1)	12 (4.5)
Headache	14 (2.5)	10 (3.7)
Patients with serious adverse events*, n (%)	46 (8.4)	24 (9.0)
COPD worsening	9 (1.6)	11 (4.1)
Pneumonia	4 (0.7)	2 (0.7)
Upper respiratory tract infection, bacterial	3 (0.5)	2 (0.7)
Atrial fibrillation	3 (0.5)	0
Dyspnoea	2 (0.4)	0
Respiratory failure	2 (0.4)	0
Cardiac failure congestive	2 (0.4)	0
Myocardial infarction	2 (0.4)	1 (0.4)
Lung neoplasm	2 (0.4)	0
Syncope	2 (0.4)	0
Upper respiratory tract infection	0	2 (0.7)
Myocardial ischaemia	0	2 (0.7)
Deaths**, n (%)	3 (0.5)	3 (1.1)
Discontinuations due to adverse events, n (%)	32 (5.8)	19 (7.1)
Electrocardiographic abnormalities, n (%)		
Notable QTcF^†^	22 (4.0)	3 (1.1)
QTcF > 500 ms	0	0
Increase from baseline of 30-60 ms	59 (10.7)	21 (7.9)
Increase from baseline of > 60 ms	6 (1.1)	1 (0.4)

* Includes five patients in the NVA237 group that had a serious adverse event that occurred during the 30-day follow-up period. **Includes one patient with a co-existing history of depression in NVA237 group, who died within 30 days after discontinuing from the study due to suicide/depression. ^† ^> 450 ms for men and > 470 ms for women

QTcF: QTc interval with Fridericia's correction

The number of deaths was low in the NVA237 group (3 patients, 0.5%) and in the placebo group (3 patients, 1.1%). Two deaths in the NVA237 group occurred during the treatment period and were both due to cancer. The third NVA237 death happened during the 30-day follow-up period and was due to suicide/depression. One death in the placebo group was due to a COPD exacerbation and another was a sudden cardiovascular death. The cause of death could not be determined in one placebo recipient, who experienced abdominal pain, fever and vomiting prior to death. None of the deaths in either treatment group were suspected to be related to study treatment by the investigator.

Notable QTcF intervals (> 450 ms for males, > 470 ms for females) were reported in 4.0% of patients treated with NVA237 and 1.1% of those receiving placebo (Table 4). Changes in QTcF interval from baseline of 30-60 ms or > 60 ms occurred in 11.8% of patients receiving NVA237 and 8.2% of those receiving placebo. No patient in either treatment group had a QTcF interval > 500 ms.

## Discussion

Bronchodilation with LABAs and LAMAs plays a central role in the management of COPD. LABAs have been widely used for many years, while the first LAMA (tiotropium) became available in Europe in 2002 and in the USA and Canada in 2004 [16]. More recently, there has been interest in new LAMAs, such as NVA237 and aclidinium, which are currently in development as long-acting bronchodilators for use in the management of COPD. These new LAMAs would be particularly valuable if they could provide bronchodilation at least equivalent to that of tiotropium, with a low incidence of troublesome adverse effects. The findings of preclinical and early clinical studies, which demonstrated a favourable efficacy and safety profile of NVA237 [6-8,17], warranted further investigation in Phase III studies.

In the Phase III GLOW1 study, once-daily NVA237 resulted in statistically significant improvement in trough FEV_1 _at 12 weeks, with a treatment difference of 108 mL. A steady state for improvement in trough FEV_1 _versus placebo was achieved at the end of Day 1 and sustained throughout the study. NVA237 also resulted in statistically significant increases in TDI score that exceeded the 1 point difference considered clinically important [14] and significant improvements in SGRQ scores, with 56% patients achieving the 4-point threshold regarded as clinically significant improvement [15]. NVA237 also significantly reduced the risk of moderate or severe COPD exacerbations and was associated with a numerical reduction in the rate of exacerbations. It should be noted that a duration of 26 weeks with the current sample size does not have adequate power to detect statistically significant differences in the rate of exacerbations. Additionally, in the population studied, a majority (> 60%) of the patients had moderate COPD and < 25% of the patients had a history of COPD exacerbations prior to screening. This accounts for the overall low rate of exacerbations observations. It also makes the improvement in the COPD exacerbations observed potentially more significant, since it may be extended to all patients with COPD and not just those with severe/very severe disease and a history of frequent exacerbations. Further, the patients with moderate-to-severe COPD who were enrolled in GLOW1 had a lower rate of exacerbations prior to enrolment (21%) than in the ECLIPSE study, in which 39% of patients with moderate COPD and 52% of those with severe COPD had one or more exacerbations during the previous year [18]. This difference may be due to variations in the definition of exacerbations (in the GLOW1 study a pre-defined criteria needed to be met for an event to be classified as an exacerbation, while the ECLIPSE study had no such criteria), duration and timing of the assessment period (e.g. whether it includes the high-risk winter months), geographic location (since exacerbation frequency may be affected by weather, climate, and air pollution), and the frequency of follow-up [19].

The improvements in trough FEV_1 _versus placebo in the GLOW1 study are consistent with previous studies of NVA237 [6,7] and are similar to those seen at Week 12 in randomized, double-blind studies of tiotropium [20-27]. However, such comparisons require caution due to differences between the studies (for example, patients enrolled in the tiotropium studies had more severely impaired lung function). Several studies of tiotropium have reported a similar percentage (40-60%) of patients achieving a clinically significant improvement in SGRQ scores as in the GLOW1 study [21,22,27-30]. Recent studies with tiotropium have also shown improvements in exacerbations, with a significant delay in time to first exacerbation and time to first hospitalization after an exacerbation [29,31]. NVA237, in the GLOW1 study, showed similar results, with a significantly prolonged time to first moderate/severe exacerbation and severe exacerbations leading to hospitalization. In these respects, NVA237 appears to produce effects which are comparable to tiotropium. However, in other studies tiotropium has been shown to reach a steady state for trough FEV_1 _only by Day 7 [4,32], compared to Day 1 with NVA237. Thus, NVA237 has a quicker time to steady state in addition to its faster onset of action [7]. Also, tiotropium has been shown in some studies [21,28] to result in clinically significant improvements in TDI score in a lower percentage of patients (45%) than NVA237 in the current study (62%).

Patients receiving NVA237 in GLOW1 had a numerically higher frequency of notable QTcF intervals (4.0% of patients), compared with placebo (1.1%). However, no patient in either treatment group had a QTcF interval > 500 ms and the overall results from the study indicated that NVA237 had a good safety profile, with a low frequency of cardiac AEs. Cardiovascular AEs of LAMAs result from blockade of M2 receptors, which are thought to modulate pacemaker activity, atrioventricular conduction and contraction force [33]. The favourable cardiac safety profile of NVA237 may therefore result from its high affinity for M3 receptors and low affinity for M2 receptors, and also from its faster dissociation from the M2 receptor than from the M3 receptor [34,35].

Hyperinflation, the main contributor to dyspnoea and reduced exercise tolerance (dynamic hyperinflation), is closely associated with IC [36,37]. An increase in IC after bronchodilator administration signifies a reduction in hyperinflation, which may translate to a reduction in dyspnoea and improved exercise tolerance. In the current study, IC for NVA237 was higher than baseline values at all timepoints (25 min, 1 h 55 min, 3 h 55 min and 23 h 40 min post dose), and was significantly higher than placebo (p < 0.001) in the FAS. It can be hypothesized that the increase in IC with NVA237 allowed for greater expansion to tidal volume and contributed to the reduction in dyspnoea. This observation offers an opportunity to further explore the effect of NVA237 on dyspnoea and exercise capacity.

## Conclusion

The results from the GLOW1 study showed that once-daily treatment with NVA237 resulted in significant improvements in FEV_1 _and health-related quality of life, and significant reductions in dyspnoea, risk of exacerbations and rescue medication use. Improvements in FEV_1 _were rapid, apparent within 5 minutes of dosing on Day 1 of treatment, and were sustained throughout a 24-hour period from Day 1 up to Week 26. In addition, NVA237 was generally safe and well tolerated, with a low incidence of adverse effects typically associated with antimuscarinic agents.

## Competing interests

AD has received research, consulting and lecturing fees from GlaxoSmithKline, Sepracor, Schering Plough, Altana, Methapharma, AstraZeneca, ONO Pharma, Merck Canada, Forest Laboratories, Novartis Canada/USA, Boehringer Ingelheim (Canada) Ltd, Pfizer Canada, SkyePharma, and KOS Pharmaceuticals. GTF has performed research funded by Novartis and received honoraria for participation in advisory panels pertaining to various COPD medications for Novartis Pharma AG. JAvN has received research support from Boehringer Ingelheim, Chiesi, Novartis and GlaxoSmithKline. KH is a consultant to Boehringer Ingelheim (Japan), AstraZeneca (Japan), and Novartis (Japan). CM, RH, YL, DB and TO are employees of Novartis Pharma AG.

## Authors' contributions

All authors had full access to the data and read and approved the final manuscript. AD was involved in acquisition of data, analysis and interpretation of data, drafting the manuscript and revising it critically for important intellectual content and provided final approval of the version to be published. GTF made substantial contributions to the current study and publication, participating in the study process, analysis of data, development and revisions of the manuscript and has approved the final manuscript draft. JAvN was involved in the interpretation of data and critical reading and revision of the draft manuscript, and final approval of the manuscript. KH made important contributions to this study, participating in the interpretation for the acquired data, development and critical reading of the draft manuscript, and final approval of the manuscript. CM developed the design, concept of the study and analysis and participated in the interpretation of the study. RH was the clinical study manager and participated in the interpretation of the data. YL contributed to the design of the study and carried out the statistical analysis. DB conceived of the study, participated in its design and contributed to its interpretation. TO participated in the development of the design and concept of the study and in the interpretation of the data.
